# Analytical Validation of MSD S‐PLEX Neurology Panel 1

**DOI:** 10.1002/alz.092387

**Published:** 2025-01-09

**Authors:** Catherine Demos, Nikhil Padmanabhan, Rachel Cohen, Jermaine Brown, Taron Gorham, Jack McBride, Sia Sesay, Sol M Rivera Valez, Allan Barnes, Jun Yang, David Graham, Allen D Everett, Anu Mathew, Martin Stengelin, George Sigal, Jacob N Wohlstadter

**Affiliations:** ^1^ Meso Scale Diagnostics, LLC., Rockville, MD USA; ^2^ Molecular Determinants Core Johns Hopkins All Children's Hospital, Saint Petersburg, FL USA; ^3^ Johns Hopkins University School of Medicine, Baltimore, MD USA

## Abstract

**Background:**

Three blood‐based biomarkers of neurological injury—glial fibrillary acidic protein (GFAP), neurofilament light (Nf‐L), and Tau—have emerged as promising biomarkers of neurological disorders and injuries such as hypoxic‐ischemic encephalopathy (HIE), traumatic brain injury, and Alzheimer’s disease (AD). The low levels of GFAP, Nf‐L, and Tau in serum and plasma require highly sensitive assays to detect them. Here, we report the analytical validation of an ultrasensitive, electrochemiluminescence‐based, multiplexed immunoassay for neurological biomarker assessment.

**Method:**

The MSD S‐PLEX Neurology Panel 1 kit uses ultrasensitive S‐PLEX assay technology to simultaneously measure GFAP, Nf‐L, and Tau (total) in a 96‐well plate format. Analytical validation was performed in a series of studies based in part on Clinical and Laboratory Standards Institute guidelines to evaluate precision, dilution linearity, interference screening, detection capability, stability, spike recovery, and cross reactivity. Reproducibility was performed to assess precision across three different laboratories at MSD, Johns Hopkins University, and All Children’s Hospital using three independent reagent lots and an identical sample set.

**Result:**

In this study, the assay used 5 μL of a sample per replicate. This differs from the commercial protocol for this panel because of the extremely high concentrations observed in HIE samples. The quantifiable range of the assay was 2.4–4,200 pg/mL for GFAP, 7.6–10,000 pg/mL for Nf‐L, and 0.34–740 pg/mL for Tau. A set of 15 common interfering substances was screened, and none showed interference exceeding 18%. Dilution of spiked samples up to 256‐fold recovered at 80‐120% of the expected value for all analytes. Precision error was calculated using identical samples across 3 independent laboratories and 3 production lots of the panel (180 measurements) demonstrating excellent reproducibility. The samples included adult and umbilical cord serum and plasma, samples containing human anti‐mouse antibodies, anti‐nuclear antibodies, and clinical samples, including those from subjects with AD.

**Conclusion:**

The MSD S‐PLEX Neurology Panel 1 kit provides a new, analytically validated tool for assessing human GFAP, Nf‐L, and Tau. The kit is sufficiently sensitive to detect these analytes in normal serum and plasma and its dynamic range is capable of quantifying elevated levels found in target neurological disorders.

